# Opportunistic pathogen *Candida albicans* elicits a temporal response in primary human mast cells

**DOI:** 10.1038/srep12287

**Published:** 2015-07-20

**Authors:** José Pedro Lopes, Marios Stylianou, Gunnar Nilsson, Constantin F. Urban

**Affiliations:** 1 Department of Clinical Microbiology, Umeå University, Umeå, Sweden; 2 Umeå Centre for Microbial Research (UCMR), Umeå, Sweden; 3 The Laboratory for Molecular Infection Medicine Sweden (MIMS), Umeå, Sweden; 4 Department of Medicine, Karolinska Institutet and Karolinska University Hospital, Stockholm, Sweden

## Abstract

Immunosuppressed patients are frequently afflicted with severe mycoses caused by opportunistic fungal pathogens. Besides being a commensal, colonizing predominantly skin and mucosal surfaces, *Candida albicans* is the most common human fungal pathogen. Mast cells are present in tissues prone to fungal colonization being expectedly among the first immune cells to get into contact with *C. albicans*. However, mast cell-fungus interaction remains a neglected area of study. Here we show that human mast cells mounted specific responses towards *C. albicans*. Collectively, mast cell responses included the launch of initial, intermediate and late phase components determined by the secretion of granular proteins and cytokines. Initially mast cells reduced fungal viability and occasionally internalized yeasts. *C. albicans* could evade ingestion by intracellular growth leading to cellular death. Furthermore, secreted factors in the supernatants of infected cells recruited neutrophils, but not monocytes. Late stages were marked by the release of cytokines that are known to be anti-inflammatory suggesting a modulation of initial responses. *C. albicans*-infected mast cells formed extracellular DNA traps, which ensnared but did not kill the fungus. Our results suggest that mast cells serve as tissue sentinels modulating antifungal immune responses during *C. albicans* infection. Consequently, these findings open new doors for understanding fungal pathogenicity.

Severe mycoses are rising in modern health care, mainly due to the use of catheters and immunosuppressive treatments^1^. The most prevalent fungal pathogen^2^, *Candida albicans* is also part of the human commensal flora. *C. albicans* commensally colonizes the gastrointestinal, urogenital, oral-nasal cavity and skin. When host immunity is suppressed, *C. albicans* can disseminate to non-commensal niches, resulting in hazardous colonization and invasive disease. *C.albicans*-associated mycoses have an annual prevalence of 300 000 with an associated mortality up to 55% in European intensive care units^3^,^4^. Due to their distribution in tissues facing external surfaces mast cells are among the first immune cells to get in contact with *C. albicans*.

Mast cells are tissue-dwelling cells derived from hematopoietic progenitors. These cells migrate from the blood to the skin, airways or the gastrointestinal tract where final differentiation is induced by surrounding structural cells. Mast cells are known for triggering hypersensitivity reactions at the body interfaces with external environments. They respond to stimuli by rapidly degranulating their cytoplasmic vesicles leading to selective and differential mobilization of granule contents into the milieu.

The role of mast cells in asthma and inflammatory disorders is an intensively active area of research, however, comparably little is known about the role of these cells in host defence^5^. Mast cells have been shown to participate in the killing of bacteria^6^,^7^,^8^, whereas their antifungal defence response is virtually unexplored^9^,^10^,^11^,^12^. Interestingly, mast cells express molecules, for instance inflammatory cytokines, myeloid-attracting chemokines, and pattern recognition receptors that were demonstrated to be involved in antifungal responses in other cells. Toll like receptors (TLRs), such as TLR2 and TLR4 are in addition to mediate responses against bacteria^13^,^14^,^15^ well-established contributors for detection and clearance of fungi^16^,^17^. Activation of C-type lectin receptors, such as dectin 1, by fungal components also results in mast cell activation^18^,^19^. This suggests a possible involvement of mast cells in antifungal immunity. Specific ligands for such receptors can activate mast cells causing release of reactive oxygen species^20^ and different vasoactive mediators, for instance histamine, prostaglandins, leukotrienes, and tryptase, which are injurious to the microbes^21^. While most of these mediators promote inflammation, they are in addition responsible to recruit other immune cells^22^,^23^.

We chose *C. albicans* to study fungal-mast cell interactions, since *C. albicans* is a commensal and a frequent human pathogen. This dual role enables a more detailed understanding of fungal pathogenicity, innate immune response and immune tolerance. We found that human mast cells have a versatile and timed response upon fungal encounter. Mast cells first degranulated β-hexosaminidase and were able to transiently reduce 30% of *C. albicans* viability up to 3 h post infection. In intermediate responses mast cells released pro-inflammatory cytokines, such as interleukin-8 (IL-8) and supernatants of *C. albicans*-infected mast cells were chemoattractive to neutrophils. In late responses mast cells secreted IL-16 and anti-inflammatory IL-1ra and released mast cell extracellular traps (MCETs) that ensnared, but probably did not kill *C. albicans*. In addition, the fungus could cause mast cell death by different mechanisms.

Ultimately, our work contributes to the understanding of the role of mast cells in modulating the innate immune response against opportunistic pathogenic fungi.

## Results

### *C. albicans* induced rapid degranulation in mast cells

**Figure 1:**
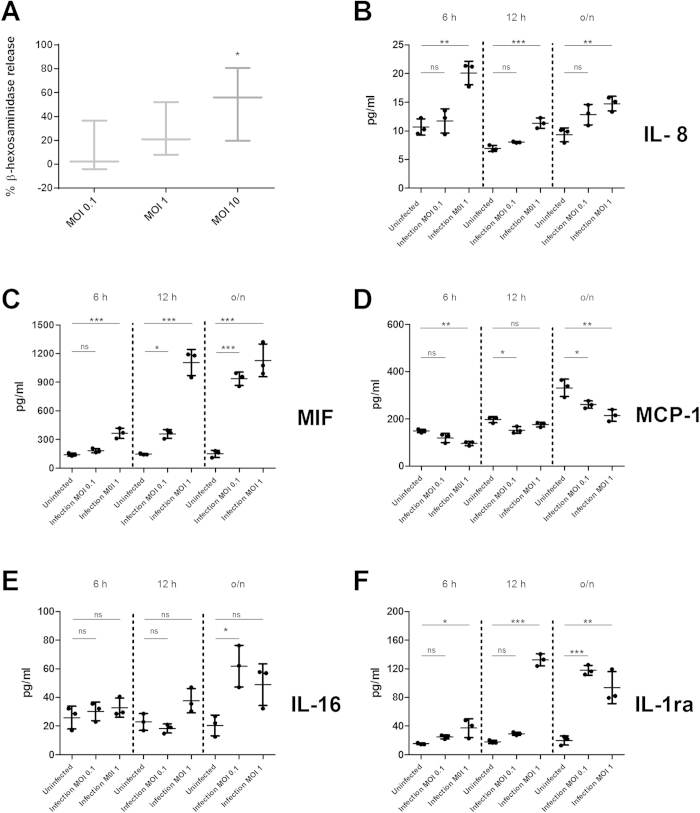
*C. albicans* induced mast cell degranulation and cytokine release in a MOI-dependent manner. (**A**) HMC-1 cells were infected with opsonized *C. albicans* yeasts (MOI 0.1, 1 and 10) for 1 hour, after which ß-hexosaminidase release was measured from supernatants of infection. (**B**–**F**) Shown are 5 cytokines at 6, 12 and 24 h post infection that were released differentially from different supernatants of mast cells infected with *C. albicans* (MOI 0.1 and 1) or of mast cells left uninfected. ß–hexosaminidase percentage release was defined by the amount of ß-hexosaminidase release from infected cell divided by spontaneous ß-hexosaminidase release from uninfected cells (% of ß –hexosaminidase release/% of ß –hexosaminidase control). Significance for (**A–F**) was analysed by Tukey one-way ANOVA *P ≤ 0.05. Data are presented as means of n = 4 (4) ± SD (ß-hexosaminidase release assay) and n = 3 (3) ± SD (Cytokine Multiplex).

Mast cells contain large amounts of enzymes in their granules^21^, particularly proteases or lysosomal enzymes like β–hexosaminidase^24^. These enzymes are involved in inflammation onset^25^,^26^ and in defence against microbes^27^,^28^,^29^. Degranulation is therefore a putative mechanism mast cells may employ to respond to *C. albicans* infection. Therefore, we measured β-hexosaminidase, a routinely used marker for mast cell degranulation, during infection of mast cells with *C. albicans.* Indeed, mast cells degranulated and released β-hexosaminidase in response to *C. albicans* after 1 h of infection in a dose-dependent manner (Fig. 1A). This indicates that mast cells recognized the fungus and mounted an early and direct response.

### Mast cells mounted a unique cytokine response upon *C. albicans* infection

To test mast cell immune modulatory responses we infected human mast cell line-1 (HMC-1) cells with *C. albicans* and subsequently analysed culture supernatants for presence of cytokines. We found 5 cytokines that were differentially released from mast cells in a time-dependent manner following infection with *C. albicans*. An early cytokine response (6 h post infection) involved release of IL-8, a strong neutrophil chemoattractant (Fig. 1B). Comparably, cord blood-derived mast cells released similar amounts of IL-8 upon *C. albicans* infection (Fig. S1).

Upon *C. albicans* stimulation, mast cells additionally secreted macrophage migration inhibitory factor (MIF), a pro-inflammatory, stress-response cytokine crucial for sustaining an inflammatory milieu (Fig. 1C)^30^. Interestingly, secretion of monocyte chemoattractant protein 1 (MCP-1), one of the key chemokines inducing migration and infiltration of monocytes/macrophages was not released (Fig. 1D). Mast cells therefore are likely to contribute to neutrophil, but not to macrophage recruitment upon *C. albicans* infection. At later time points (12 and 24 h), the cytokine profile revealed the release of IL-16, a chemokine linked to chemoattraction of CD4^+^ T lymphocytes^31^ (Fig. 1E). The pro-inflammatory cytokine response at early time points post infection seems to be counteracted by release of the anti-inflammatory cytokine IL-1ra at 24 h (Fig. 1F). Taken together, these data suggests that secretion of pro- and anti-inflammatory cytokines was a controlled process that was influenced by different stages of the infection.

### Human neutrophils but not monocytes were chemoattracted towards *C. albicans*-infected mast cells

**Figure 2:**
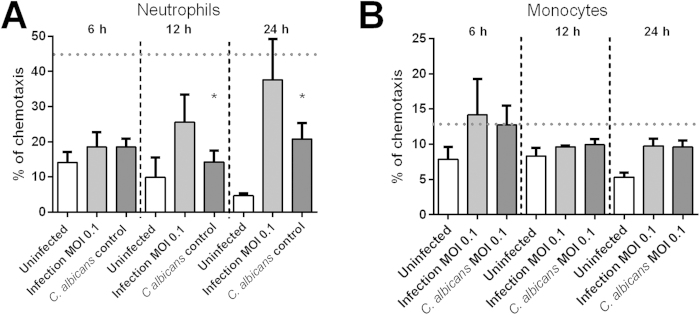
Neutrophils but not monocytes differentially migrated in response to supernatants from mast cells infected with *C. albicans*. Supernatants collected from *C. albicans*-infected mast cells (MOI 0.1) at 6 h, 12 h and overnight infection were used as chemoattractants to neutrophils and monocytes in a transwell system. End-point cell migration was plotted per condition, per time as ratio of migrated cells using as 100% control cells added to the lower compartment without inserted transwell system. **(A)** Neutrophil migration is increased over time towards supernatants of infection but not to *C. albicans* and HMC-1 alone (controls). **(B)** Monocytes show no significant chemoattraction towards supernatants of infected mast cells. Variations between neutrophil or monocyte migration over time towards supernatants of infection and *C. albicans* control were analysed for statistical significance using a one-way ANOVA with Tukey post-test. As positive control for migration we used fMLP causing chemotaxis significantly above background of approximately 45% after 30 min for neutrophils and 13% after 90 min for monocytes. These values are indicated as a horizontal, dashed line in the graphs of the figure. Data are presented as means of n = 5 (3) ± SD.

As some of the chemokines from our multiplex screening are relevant in host immune cell recruitment, we next tested the chemoattractive potential of supernatants from *C. albicans*-infected mast cells towards neutrophils and monocytes. Mast cells were infected for three time points and supernatants harvested. The chemotactic potential of the supernatants was tested using fluorescently labelled neutrophils in a transwell system (Fig. 2A). Chemoattractant fMLF was used as positive control^32^.

Notably we found, that *C. albicans*-infected supernatants induced migration of neutrophils similar to fMLF, whereas *C. albicans* alone or uninfected mast cells induced significantly lower neutrophil migration. Chemotaxis was significantly above controls with supernatants collected after 12 h or longer. The migration assay shows slightly delayed neutrophil chemotaxis compared to the cytokine release assay revealing increased IL-8 already after 6 h of infection (Fig. 1B). However, neutrophil migration might be influenced by other chemokines that were not analysed with the multiplex assay used. On the other hand analysis of monocyte chemotaxis corroborated the cytokine multiplex results. Monocyte-attractant chemokine MCP-1 was secreted by uninfected mast cells, however not induced upon *C. albicans* infection of mast cells (Fig. 1D). In contrast, monocyte-inhibitory MIF was increasingly released by infected mast cells over time (Fig. 1C).

Taken together, we confirmed our findings regarding mast cells cytokine-release following fungal infection by a functional migration assay revealing that mast cells secrete neutrophil chemoattractants.

### Despite releasing extracellular traps mast cells only transiently control *C. albicans* viability

**Figure 3:**
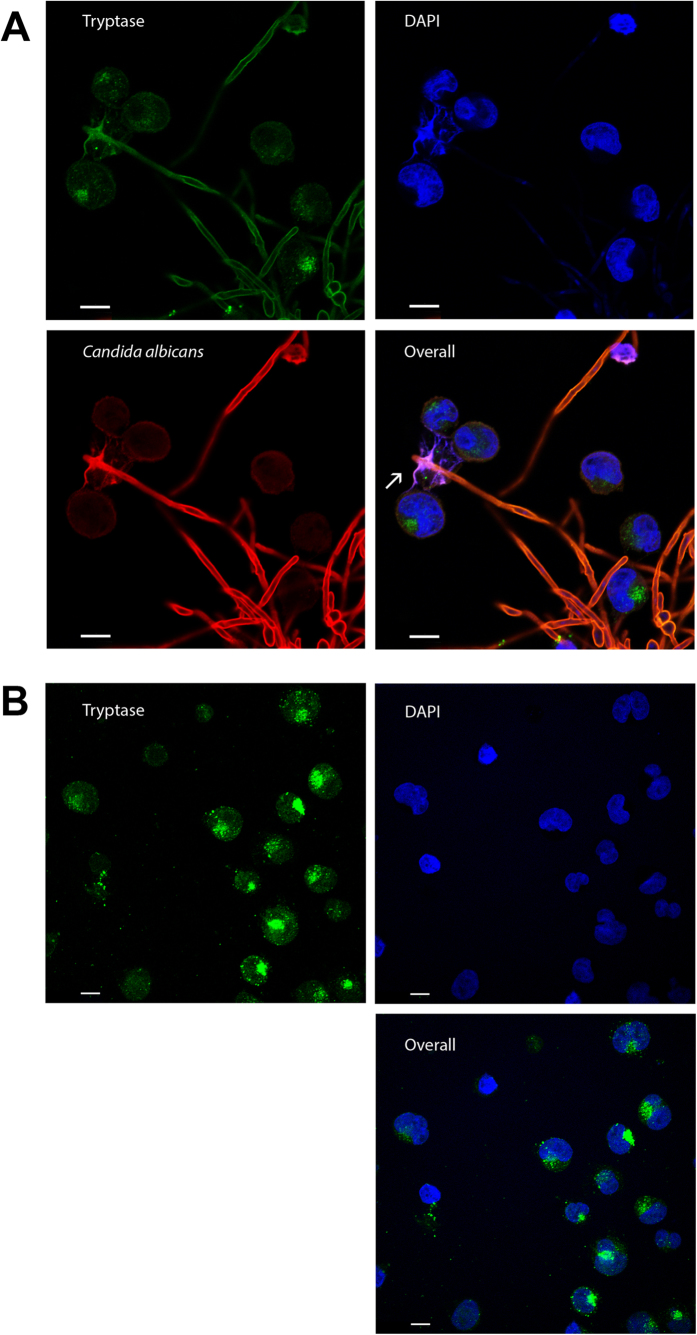
*C. albicans* induced MCETs in a time-dependent manner. Mast cells were infected for 6 h with *C. albicans* with an MOI 0.1 (**A**) or left uninfected (**B**). Shown are representative micrographs of indirect immunofluorescence from fixed and permeabilized samples with DNA (blue), mast cell tryptase (green) as well as *C. albicans* (red) stained samples. MCETs were identified by co-localization of extracellular laminar DNA with tryptase immunostaining (arrows). Scale bars, 10 μm.

**Figure 4:**
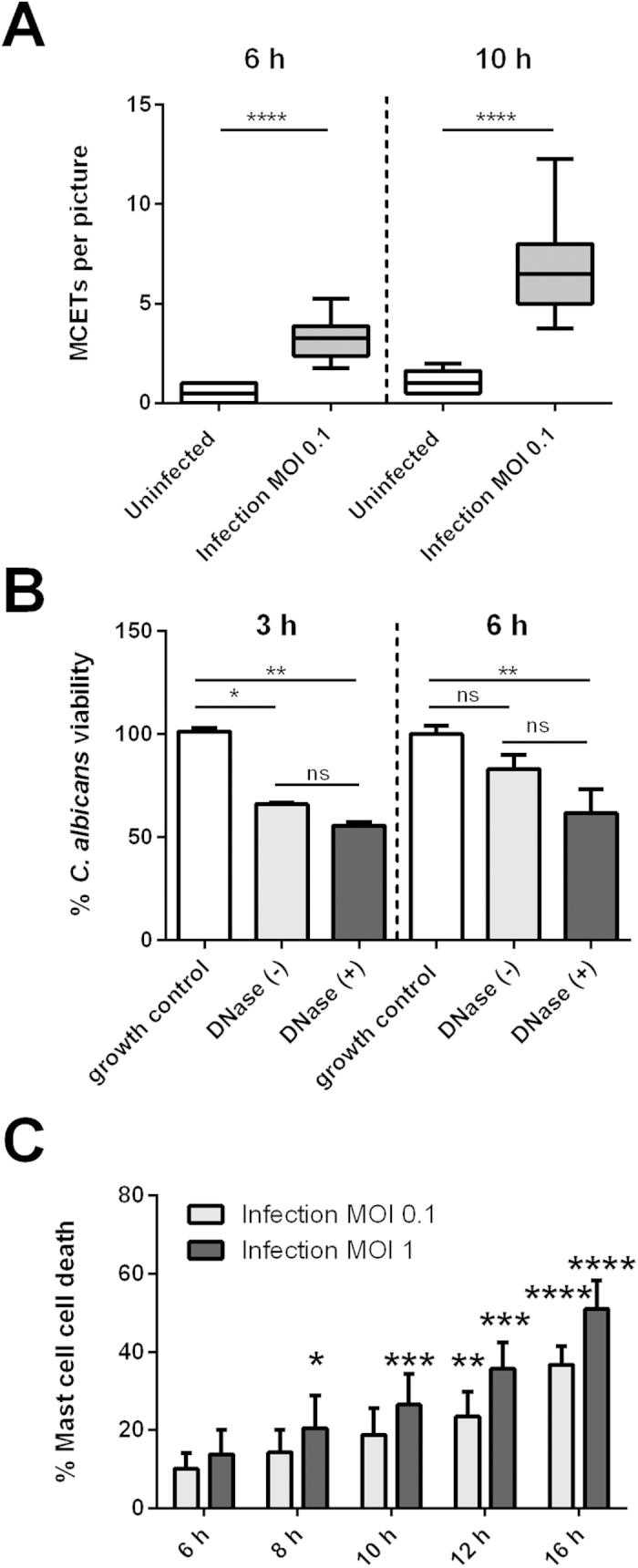
Quantification of cellular death and antifungal activity of mast cells. (**A**) The graph depicts the relative amount of MCETs per micrograph of *C. albicans*-infected mast cells (MOI 0.1) as compared to the uninfected mast cell control at two different time points. At both time points analysed (6 h and 10 h) the variation between MCETs compared to uninfected samples was analysed for statistical significance. (**B**) The graph represents the viability of fungal cells after normalization to the biological replicates 100% growth control. *C. albicans* viability is reduced in mast cell infection (MOI 1) up to 3 h. (**C**) *C. albicans*-induced mast cell death in a time and dose-dependent manner as determined with Sytox green. The Y-axis represents the relative amount of dead cells after normalization to the mast cell lysis control. (**A**) Significance was analysed by t-test and by Tukey one-way ANOVA (**B**) *P ≤ 0.05. (**B**) Data are presented as means of at least six replicates (**A**) and n = 3 (6) ± SD. (**C**) For cell death significance was analysed by Bonferroni two-way ANOVA *P ≤ 0.05 comparing to the mast cell uninfected control at each time point. Data represents n= 4 (5) ±SD.

The release of extracellular DNA traps is part of the innate immune response to infection. Extracellular traps have been observed in mast cells in response to auto-inflammatory skin diseases and upon bacterial infection^6^,^14^,^33^. Here, we investigated the potential release of MCETs in response to fungi. After 6 h, *C. albicans*-infected mast cells released MCETs composed of DNA and granular proteins ensnaring the fungus (Fig. 3A arrow, Movie S1), whereas these structures were absent in uninfected control (Fig. 3B). Primary mast cells similarly released extracellular traps upon *C. albicans* stimulation (Fig. S2A, arrow). Quantification of MCETs in a blinded fashion revealed that extracellular trap formation increased over time being significantly different from uninfected controls, but rarely exceeded 5% of the total amount of cells (Fig. 4A). Infection with higher MOIs led to MCET formation (Fig. S2B, arrow) without further increase in number (data not shown).

To account for mast cell antifungal activity, we quantified ATP levels correlating with the presence of metabolically active fungal cells. Within 3 h of incubation *C. albicans* viability was reduced by 30%. However, this antifungal effect was transient and declined after 6 h, possibly due to fungal overgrowth.

In a similar assay, we tested the antifungal activity of MCETs by degrading their DNA backbone with DNase. The nuclease was added before infection of mast cells with *C. albicans* and not removed during the whole assay to ensure degradation of any emerging MCET. We did not observe a significant difference in fungal viability in the presence of DNase as compared to samples without DNase (Fig. 4B). We conclude that mast cells display moderate antifungal activity, which appears to be MCET-independent. The traps, nevertheless, ensnared *C. albicans* (Fig. 3A).

### *C. albicans* potently triggered mast cell death

To quantify mast cell death induced by *C. albicans* including MCET formation with a microscopy-independent method we used a microplate-based fluorescence assay with Sytox Green. The dye is impermeable for intact membranes staining DNA in the extracellular space and in dead cells. Cellular death was calculated as percentage of lysis control (Materials and Methods). Remarkably, mast cell infection with *C. albicans* resulted in 50% mast cell death after 10 h (Fig. 4C). Sytox-based cellular death quantification was confirmed by detection of lactate dehydrogenase (LDH) release into culture supernatants with similar results (data not shown). Similar levels of cell death were induced by *C. albicans* when cord blood-derived mast cells were infected (Fig. S3), confirming that our results are valid for primary immune cells.

This high percentage of cellular death cannot be explained by release of MCETs shown before, and therefore is governed by other mechanisms.

### Internalized *C. albicans* can outgrow from mast cells

To assess other forms of mast cell death during interaction of mast cells and *C. albicans* we used live cell microscopy.

**Figure 5:**
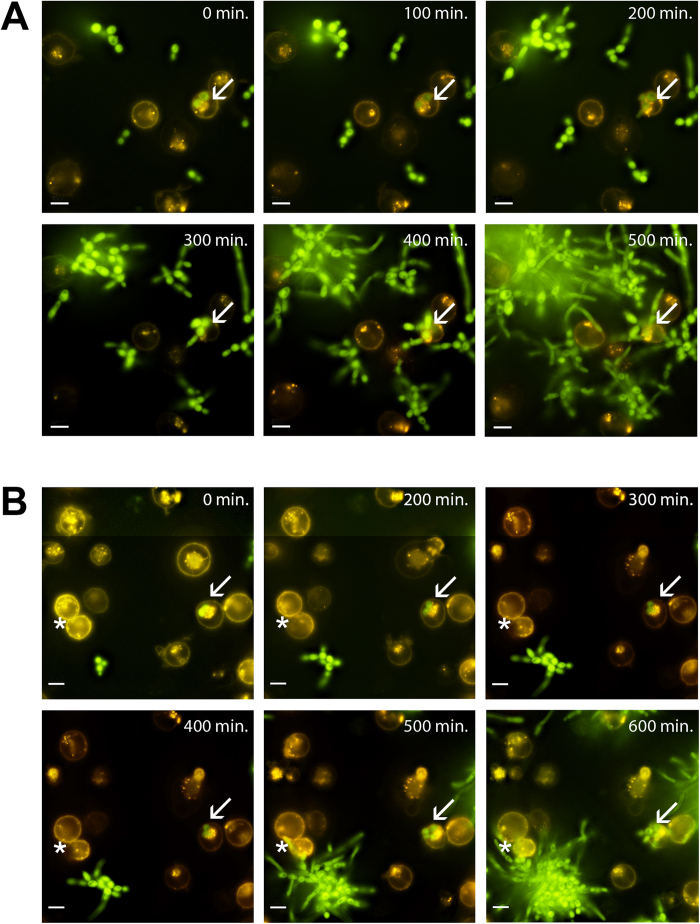
*C. albicans* internalization is followed by fungal outgrowth. Shown are images of the indicated time points for *C. albicans* infected mast cells (MOI 0.1). (**A**) Arrows show an intracellular yeast cell (GFP-expressing *C. albicans* strain *CAI4 pENO1-GFP-CyC1t*) replicating inside the mast cell (orange, membrane stain DiI) finally rupturing the plasma membrane as determined by loss of signal. An extracellular *C. albicans* hyphal tip growing towards a mast cell nudged the host cell and induced collapse of the plasma membrane (*). Complete movies are available as Movie S2 and S3 in the supplemental material. Scale bar, 10 μm.

Interestingly, we found *C. albicans* yeast cells internalized into a few mast cells as soon as 30 min after initial infection. These yeast cells were able to grow intracellularly. Whether the fungal cells promote their own uptake or are actively phagocytized by mast cells remains to be determined. We observed intracellular propagation of *C. albicans* and subsequent germination resulted in collapse of mast cell plasma membranes. (Fig. 5A, arrow; Movie S2 and S3). Inside-out growth is a rare event estimated to occur in approximately 1% of all imaged cells.

We observed collapse of the membrane of the mast cells induced by extracellular *C. albicans* hyphae that grew towards the human cell (Fig. 5B, asterisk; Movie S3). Outside-in growth is a much more common event observed in around 15–20% of all imaged cell.

The live cell microscopy revealed a plausible explanations for the high mast cell death and putative outgrowth mechanism of *C. albicans*.

## Discussion

Mast cells are crucial players in inflammatory and allergic processes. In recent years a growing body of evidence shed new light on a broader role of mast cells in host immunity^6^,^24^,^34^. Opportunistic fungal pathogens are an increasing burden to modern health care, due to a growing number of immunocompromised individuals as well as emergence of treatment-refractory strains^35^. Commensal and ubiquitous opportunistic fungi colonize or enter the body via mast cell-rich tissues. A role of mast cells in antifungal immunity is therefore likely, however, virtually unexplored.

We hypothesized that *C. albicans*, one of the most frequent microbial pathogens and a common commensal on mucosal surfaces is recognized by human mast cells. Using an *in vitro* model we studied mast cells and *C. albicans* interactions using different approaches to qualitatively and quantitatively access this interplay. In this study we show that both primary- and cell line derived-mast cells responded to *C. albicans* in a specific and organized manner. The responses involved different stages, including initial, rapid degranulation followed by temporal release of cytokines that mediated cell recruitment and fungal recognition.

We demonstrated that mast cells initially affected *C. albicans* viability. The effect was temporary and decreased over a 6 h period, opposing the description of Trevisan *et al.*^36^ who showed that rat peritoneal mast cells permanently killed *C. albicans* . The same group additionally reported rat peritoneal mast cell degranulation towards unopsonized *C. albicans*^36^. Our study shows that mast cells degranulated more intensely towards opsonized *C. albicans* cells. Both, the previous and our study oppose a third using a rat-derived RBL-2H3 cell line^37^, in which the authors could not identify degranulation. Thus, we assume that these diverging results stem from differences in mast cell origin and maturation and from the fact that humans are natural hosts for *C. albicans*, whereas rats are not.

Human mast cells create an inflammatory environment by secreting a specific cytokine pattern during *C. albicans* challenge. Recruitment of innate immune cells, such as tissue macrophages and circulating neutrophils, is essential for clearing of fungal infection. We identified neutrophil-attracting chemokine IL-8 released by infected mast cells and translated the findings into a functional assay showing that human neutrophils migrate to infection supernatants. IL-8 release and the resulting recruitment of neutrophils is a known feature in mucosal tissues during candidiasis^38^, whereas the contribution of mast cells to this environment was not described. Importantly, IL-8 release was confirmed with primary mast cells, highlighting that the cytokine release was not restricted to the cell line HMC-1. The umbilical relation between mast cells and neutrophils has been demonstrated in an earlier report^39^ where injection of compound 48/80 in the mouse skin evoked an acute inflammatory reaction leading to a dose-dependent elevation of leukocyte numbers 4 h after challenge. Additional reports^40^,^41^ have also shown that tissue-resident mast cells control the early stage of neutrophil recruitment during tissue inflammation. In a 2015 report by Weber and colleagues^42^, mast cell deficiency inhibited neutrophil accumulation at the site of sensitization in a model of human allergic contact dermatitis.

In our experimental setting *C. albicans*-infected mast cells contribute to macrophage recruitment to a minor extent as supported by the secretion of inhibitory factor MIF, the diminished released of macrophage-chemoattractant MCP-1, and the absence of chemotattractant activity of the mast cell infection supernatants towards monocytes. The inflammatory response was changed at later time by secretion of anti-inflammatory mediators and chemokines for adaptive immune cell recruitment. In an attempt to demonstrate chemotaxis of T-cells towards mast cell infection supernatants collected from late time points we observed high spontaneous migration of Jurkat cells. This is consistent with a previous report^43^ and likely to mask T-cell migration towards specific chemoattractants present in our supernatants. In accordance to this notion, CD4^+^ T-cell-mediated responses have been observed in models of fungal infection where antibiotic use caused *C. albicans* overgrowth and increased levels of mast cells proliferation^44^.

Extracellular trap formation was first described in neutrophils by Brinkmann *et al.*^45^ and later in several other immune cells^46^,^47^,^48^. Extracellular traps act as danger signals in infection or inflammatory diseases, as extracellular release of proteases and other injurious cell constituents can exacerbate inflammatory processes^33^,^49^. In 2008 von Kockritz-Blickwede *et al.*^6^ showed that mast cells release chromatin decorated with granule proteins in extracellular filaments that bind to and kill bacteria. Interestingly, MCET release appears to be a mechanism of immune defence present in the mast cell toolbox against fungal pathogens as both primary cells and HMC-1 release MCETs upon *C. albicans* stimulation. In contrast to bacteria^6^,^14^, however, *C. albicans* viability was not affected by the mast cell-derived DNA fibbers and thus MCETs rather contribute to physical restriction of fungal pathogens.

Since MCET release alone was not sufficient to explain high mast cell death rates during infection, we decided to assess other forms of cellular death by live cell microscopy. We found that *C. albicans* could be internalized into mast cells, which seemed to occur rarely. The live cell imaging setup used does not unambiguously allow determining whether a *Candida* cell is within or attached to a mast cell.

Nevertheless, the growth of *C. albicans* hyphae towards and until attachment to mast cells from the outside (outside-in growth) resulted in loss of membrane dye and thus integrity of the host cell membrane, clearly demonstrating that mast cells are ruptured by *C. albicans.* This, at least in part, explains the high mast cell death during interaction with *C. albicans*, because it is a more frequent event (in 15–20% of the cells). *C. albicans* can induce cell death in host cells. For instance, mucosal spread of *C. albicans* involves the adherence to and invasion of epithelial cells resulting in tissue damage^50^. In macrophages, for instance, both yeast^51^ and hyphae^52^ can promote intracellular escape and host cell death. However, the exact mechanism and the *in vivo* relevance of *C. albicans*-induced mast cell death needs to be determined in further studies.

**Figure 6:**
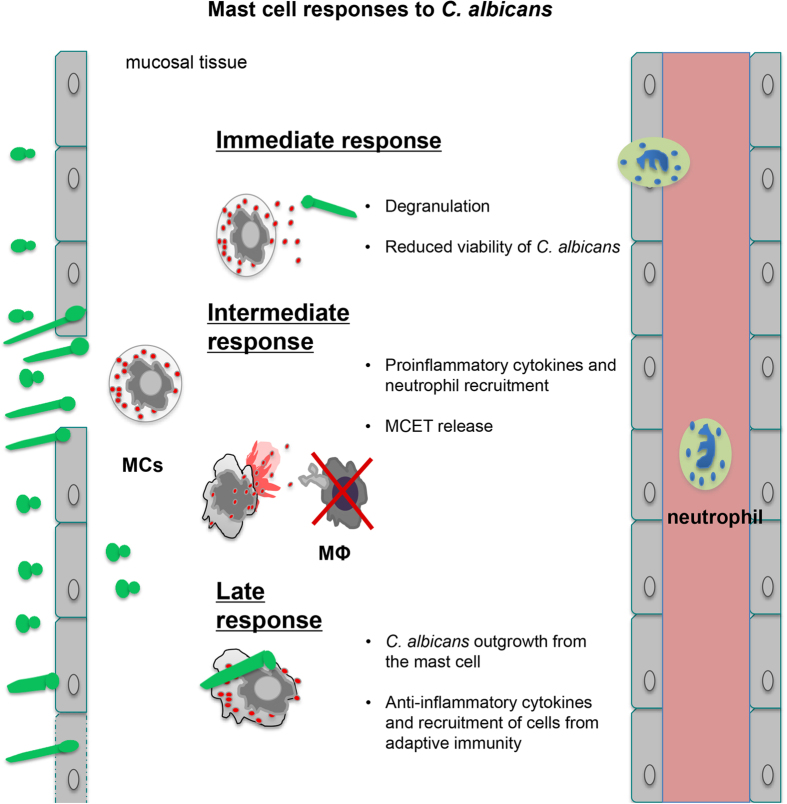
Summary of mast cell and *C. albicans* interaction. Our findings indicate that mast cells specifically respond to *C. albicans* by degranulation, secretion of cytokines and chemokines, internalization of *C. albicans* and the release of MCETs. Interestingly, the processes seem to be organised and thus can be divided into three time periods: Initial (up to 3 h), intermediate (3 h to 12 h), and late responses (>12 h).

The described processes (Fig. 6) strongly suggest a role of mast cells as sentinels during the initial phase of fungal infection. Mast cells launched an immune response that contributes to elimination of the encountered fungal pathogen either directly by mast cell-dependent contact or indirectly by the recruitment of other immune cells. Nevertheless further investigation *in vivo* will help to access the contribution of mast cells to antifungal defence.

In spite of our findings that mast cells mounted a strong immune response against *C. albicans,* allergic reactions in humans are rarely triggered by this fungus alluding to a tolerance mechanism of the host^53^. Knowledge about this tolerance mechanism could help to improve treatment of common fungal-mediated asthma.

## Methods

### *C. albicans* culture conditions

*C. albicans* strains used in this study were: clinical isolate SC5314 (wild type)^54^ and for live cell imaging *C. albicans* (CAI4) ENO1 promoter GFP fusion.

For all experiments, fungal cultures were inoculated overnight in synthetic complete dropout medium +2% glucose (SC) at 30 °C. A fresh subculture was inoculated in SC medium for 3 h at a starting cell number of 1 × 10^7^ cells/ml followed by 30 min opsonisation using pooled and heat-inactivated human serum 2% final concentration. *C. albicans* cells were centrifuged and re-suspended in PBS. The number of yeast cells was adjusted to the desired multiplicity of infection (MOI) prior to infection.

### Plasmids and transformation of *C. albicans*

For this study, we generated a constitutively GFP- expressing *C. albicans* strain. A *C. albicans*-specific GFP^55^ (Genscript) was integrated into pUC57 via PstI and XbaI restriction sites for further integration into pCaEXP^56^. pCaEXP was linearized via StuI restriction digestion in the RP10 gene for integration of pENO1-GFP in strain CAI4^57^, resulting in a *C. albicans* strain *(CAI4 pENO1-GFP-CyC1t)* constitutively expressing GFP^58^. Positive transformants were selected by plating on SC–uridine medium and the insertion of the GFP construct in the *C. albicans* genome was confirmed by sequencing.

### Cell culture conditions

Cells from the human mast cell line HMC-1^59^ and U937 cells (human monocytic cell line) were maintained in RPMI 1640-GlutaMax (Life Technologies) supplemented with 10% fetal calf serum, 100 U/ml penicillin and 100 μg/ml streptomycin (Lonza). Cord blood-derived mast cells (CBMCs) from CD34^+^ selected cells (Miltenyi Biotec) were cultivated in StemPro-34 SFM medium (Invitrogen) supplemented with 100 ng/mL recombinant human SCF (hSCF, Peprotech) and 10 ng/mL human IL-6 (Peprotech) (first week: additionally 10 ng/ml human IL-3) for 4 weeks and then maintained as previously described^60^. All cells were kept at 37 °C, 5% CO_2_. Medium was refreshed every 2–3 days. The culture was routinely checked for Mycoplasma contamination (MycoAlert, mycoplasma detection kit, Invitrogen).

Prior to any assays, mast cells were primed with 25 nM PMA (12-myristate-13-acetate, Sigma-Aldrich) for 15 min at 3 °C as previously described^6^. Cells were then centrifuged for 10 min at 300 × g and re-suspended in RPMI without fetal calf serum or antibiotics for the assays.

### N-acetyl-β-D-hexosaminidase release assay

β–hexosaminidase secretion was measured according to a previous report^61^ with minor modifications. HMC-1 cells (1 × 10^5^ cells/well) were infected, in a 96-well plate with different cell numbers of *C. albicans* (MOI 0.1, 1 and 10) or left untreated. After 1 h supernatants from technical replicates were pooled and added to new 96-well plates in triplicate.

### Mast cell cytokine release

Mast cells (1 × 10^6^ cells/well) were seeded in 24-well plates (BD Falcon) and infected at 37 °C, 5% CO_2_ with *C. albicans* at MOI 0.1, 1 or left untreated. *C. albicans* without any further additives was used as a fungal growth control. After infection, cells were centrifuged at 300 × g and supernatants collected and pooled. Debris in the mixture was further removed by centrifugation at 3000 × g for 10 min at 4 °C. The supernatants were harvested, shock-frozen in liquid nitrogen and stored at −80 °C. The cytokine levels in the supernatants from the infections and respective controls were analysed using the Bio-Plex human cytokine 27-plex and 21-plex panel (Bio-Rad Inc., USA) for the following cytokines: IL-1β, IL-1ra, IL-2, IL-4, IL-5, IL-6, IL-7, IL-8, IL-9, IL-10, IL-12, IL-13, IL-15, IL-16, IL-17, IL-18, IL-5, Eotaxin, FGF Basic, G-CSF, GM-CSF, IFN-γ, IP-10, MCP-1, MIP-1a, PDGF-BB, MIP-1β, RANTES, TNF-α, VEGF, IL-1 α, IL-2RA, IL-12, CTACK, GROα, HGF, IFN-α2, LIF, MCP-3, M-CSF, MIF, MIG, β-NGF, SCF, SCGF-β, SDF-1 α, TNF-β, TRAIL. Samples were mixed with antibody-coated beads that have a unique fluorescent intensity for the above cytokines. Anti-cytokine antibody PE-conjugated with streptavidin was added and the fluorescent signals were detected using a multiplex array reader Bio-Plex 200 System (Bio-Rad Laboratories). Raw data were initially measured as relative fluorescence intensities and then converted to cytokine concentrations based on the standard curve generated from reference concentrations supplied by the manufacturer.

Concentrations of IL-8 in supernatants of *C. albicans*-infected CBMC were measured using a human IL-8 enzyme-linked immunosorbent assay (ELISA) MAX kit (Biolegend, eBioscience USA). Primary cells were infected with *C. albicans* at MOI1 or left uninfected for 6 h. Supernatants were harvested and stored as described above.

### Chemotaxis assay for human neutrophils and monocytes

Neutrophils were harvested from blood of healthy volunteers according to the recommendations of the local ethical committee (Regionala etikprövningsnämnden i Umeå). Fully informed consent was obtained, and all investigations were conducted according to the principles stated in the Declaration of Helsinki. Neutrophils were isolated from venous blood as previously described^32^. Chemotactic migration of neutrophils and monocytes towards supernatants of mast cells infected with *C. albicans* (MOI 0.1), uninfected controls or the equivalent amount of fungal cells was measured using a transwell system as previously described^32^. Neutrophils migration was accessed for 30 min and for monocytes migration was accessed for 90 min. Mast cell infected supernatants tested were collected and stored as described for the cytokine release assay at 6 h, 12 h and 16 h.

### Immunostaining and microscopic analysis of mast cells

Mast cells (1 × 10^5^ cells/well) were seeded onto cover slips coated with 1% poly-l-lysine (Sigma-Aldrich) in 24-well plates and infected with *C. albicans* (MOI 0.1, 1). Uninfected mast cells were used as control. After 6 h cells were fixed using 2% paraformaldehyde and stored at 4 °C.

For visualization of MCETs, primary antibodies directed against human mast cell tryptase (clonal AA1, mouse, DAKO) and *C. albicans* antibody (mouse monoclonal, ProSci) diluted in blocking solution were applied overnight at 4 °C. Primary antibodies were detected with Alexa Fluor 488- and 568-conjugated secondary antibodies (Life Technologies). DNA was visualized with DAPI (4′, 6′-diamidino-2-phenylindole; Life Technologies). Specimens were mounted in Pro-Long Diamond (Life Technologies).

Imaging data were acquired using a fully motorized inverted microscope (Nikon A1R Laser Scanning Confocal Microscope) with 60 × oil immersion lens (Plan Apochromat VC; Nikon, Tokyo, Japan) under control of the NIS-Elements microscope imaging software (Nikon). Final image composition was done using Adobe Photoshop CS5 (San Jose, CA).

For live cell microscopy mast cells (2 × 10^5^ cells/well) were stained with Vybrant DiI Cell-labelling solution (Invitrogen) according to the manufacturer’s instructions and seeded into a 35-mm glass-bottom micro- well dish (MatTek, Ashland, MA, USA). Mast cells were infected with *C. albicans* strain *(CAI4 pENO1-GFP-CyC1t)* at MOI 1 and kept at cell-culture conditions throughout the measurement. Frames were captured 30 min post-infection at 60 × magnification every 10 min for a period of 16 h using the previously described microscope.

Microscopic quantification was performed using DAPI immuno-stained image samples from six biological replicates. Images analysed had 130 ± 30 cells per picture and for each infection condition a total of at least 1000 cells were analysed. The total number of cells was determined by ImageJ version 2.0. The number of cells that underwent MCET formation was scored from binary images in a blinded fashion by two trained researchers. Final scores were defined as MCETs per field of view and plotted by condition and infection end-point.

From 10 independent live cell movies the % of cells undergoing inside-out growth was determined as the ratio of [(inside-out growth)/100% total cells)]. Similar the % of cells undergoing outside-in growth was determine as the ratio of [(outside-in side growth)/100% total cells)]. In both cases a total of 80 cells was analysed.

### Fungal viability measurement

To determine the antifungal effect of mast cells we compared fungal viability in the presence of mast cells as follows: Mast cells (5 × 10^4^ cells/well) were infected with *C. albicans* at MOI 1 for 3 and 6 h in a 96-well plate coated with poly-l-lysine. The same amount of *C. albicans* served as 100% control. To test for contribution of MCETs to fungal viability we added DNaseI (Sigma Aldrich) prior to infection to one set of experiments. At the end point of the experiment, DNaseI and subsequently Triton-X100 to a final concentration of 10% were added to all wells. The medium was removed and fungal viability (ATP) was determined using CellTiter-Glo cell viability kit (Promega) in a luminometer (Tecan Infinite F200) as previously described^62^.

To normalize all values for comparable ATP signals, values were multiplied by the factor: [average of technical replicates of 100% growth control]/[average of biological replicates of 100% growth controls]. Using these normalized values, the antifungal effect was determined as the ratio of [(infected MCs–uninfected MCs)/100% growth *C. albicans*)].

To assure that any differences in cell viability were not due to loss of cells during washing we measured absorbance before triton lysis and after adding CellTiter-Glo reagent and found no notable variation.

### Cell death assay

Cellular death of mast cells was quantified using a Sytox Green-based assay as previously described for human neutrophils^32^. Mast cells (5 × 10^4^ cells/well) were seeded in a black 96-well plate in the presence of 2.5 μM Sytox Green and infected with *C. albicans* - MOI 0.1, 1- or left untreated. The same amount of *C. albicans* served as viability control whereas triton-lysed mast cells served as a reference for 100% cellular death.

### Statistics

Statistical analysis was performed using GraphPad Prism Software 6.01 (GraphPad Software, La Jolla, CA, USA).

Cytokine profile n = 3 (3), β–hexosaminidase n = 4 (4), chemotaxis n = 5 (3) and fungal viability n = 3 (6) results were analysed applying one-way ANOVA with Tukey’s post-test. ELISA results n = 2 (3), 30 min end-point chemotaxis quantification and microscopic quantification of MCETs n = 6 were analysed using t-test with Welsh correction to compare uninfected control and infection conditions in the corresponding time point. Cell death were analysed as n = 4 (5) experiments applying two-way ANOVA with Bonferroni’s post-test.

For all analyses p-values < 0.05 was considered statistically significant.

## Additional Information

**How to cite this article**: Lopes, J.P. *et al.* Opportunistic pathogen *Candida albicans* elicits a temporal response in primary human mast cells. *Sci. Rep.* **5**, 12287; doi: 10.1038/srep12287 (2015).

## Supplementary Material

Supplementary Information

Supplementary movie 1

Supplementary movie 2

Supplementary movie 3

## References

[b1] Mikulska M., Del Bono V., Ratto S. & Viscoli C. Occurrence, presentation and treatment of candidemia. Expert Rev Clin Immunol 8, 755–765, 10.1586/eci.12.52 (2012). 23167687

[b2] Patterson T. F. Advances and challenges in management of invasive mycoses. Lancet 366, 1013–1025, 10.1016/S0140-6736(05)67381-3 (2005). 16168784

[b3] Montravers P. *et al.* A multicentre study of antifungal strategies and outcome of Candida spp. peritonitis in intensive-care units. Clin Microbiol Infect 17, 1061–1067, 10.1111/j.1469-0691.2010.03360.x (2011). 20825438

[b4] Bassetti M. *et al.* Epidemiology, species distribution, antifungal susceptibility, and outcome of candidemia across five sites in Italy and Spain. J Clin Microbiol 51, 4167–4172, 10.1128/JCM.01998-13 (2013). 24108614 PMC3838046

[b5] Abraham S. N. & St John A. L. Mast cell-orchestrated immunity to pathogens. Nat Rev Immunol 10, 440–452, 10.1038/nri2782 (2010). 20498670 PMC4469150

[b6] von Kockritz-Blickwede M. *et al.* Phagocytosis-independent antimicrobial activity of mast cells by means of extracellular trap formation. Blood 111, 3070–3080, 10.1182/blood-2007-07-104018 (2008). 18182576

[b7] Cruse G. *et al.* Human lung mast cells mediate pneumococcal cell death in response to activation by pneumolysin. J Immunol 184, 7108–7115, 10.4049/jimmunol.0900802 (2010). 20483738

[b8] Rodriguez A. R. *et al.* Mast cell TLR2 signaling is crucial for effective killing of Francisella tularensis. J Immunol 188, 5604–5611, 10.4049/jimmunol.1200039 (2012). 22529298 PMC3358431

[b9] Selander C., Engblom C., Nilsson G., Scheynius A. & Andersson C. L. TLR2/MyD88-dependent and -independent activation of mast cell IgE responses by the skin commensal yeast Malassezia sympodialis. J Immunol 182, 4208–4216, 10.4049/jimmunol.0800885 (2009). 19299719

[b10] Urb M., Pouliot P., Gravelat F. N., Olivier M. & Sheppard D. C. Aspergillus fumigatus induces immunoglobulin E-independent mast cell degranulation. J Infect Dis 200, 464–472, 10.1086/600070 (2009). 19527167

[b11] Saluja R., Metz M. & Maurer M. Role and relevance of mast cells in fungal infections. Front Immunol 3, 146, 10.3389/fimmu.2012.00146 (2012). 22707950 PMC3374363

[b12] Urb M. & Sheppard D. C. The role of mast cells in the defence against pathogens. PLoS Pathog 8, e1002619, 10.1371/journal.ppat.1002619 (2012). 22577358 PMC3343118

[b13] Supajatura V. *et al.* Differential responses of mast cell Toll-like receptors 2 and 4 in allergy and innate immunity. J Clin Invest 109, 1351–1359, 10.1172/JCI14704 (2002). 12021251 PMC150977

[b14] Scheb-Wetzel M., Rohde M., Bravo A. & Goldmann O. New Insights into the Antimicrobial Effect of Mast Cells against Enterococcus faecalis. Infect Immun 82, 4496–4507, 10.1128/IAI.02114-14 (2014). 25114115 PMC4249337

[b15] Carlos D. *et al.* TLR2-dependent mast cell activation contributes to the control of Mycobacterium tuberculosis infection. Microbes Infect 11, 770–778, 10.1016/j.micinf.2009.04.025 (2009). 19442756

[b16] Inoue M. & Shinohara M. L. Clustering of pattern recognition receptors for fungal detection. PLoS Pathog 10, e1003873, 10.1371/journal.ppat.1003873 (2014). 24586145 PMC3930597

[b17] Shoham S., Huang C., Chen J. M., Golenbock D. T. & Levitz S. M. Toll-like receptor 4 mediates intracellular signaling without TNF-alpha release in response to Cryptococcus neoformans polysaccharide capsule. J Immunol 166, 4620–4626 (2001). 11254720 10.4049/jimmunol.166.7.4620

[b18] Kimura Y. *et al.* Dectin-1-mediated signaling leads to characteristic gene expressions and cytokine secretion via spleen tyrosine kinase (Syk) in rat mast cells. J Biol Chem 289, 31565–31575, 10.1074/jbc.M114.581322 (2014). 25246527 PMC4223353

[b19] Olynych T. J., Jakeman D. L. & Marshall J. S. Fungal zymosan induces leukotriene production by human mast cells through a dectin-1-dependent mechanism. J Allergy Clin Immunol 118, 837–843, 10.1016/j.jaci.2006.06.008 (2006). 17030235

[b20] Yang Z. & Marshall J. S. Zymosan treatment of mouse mast cells enhances dectin-1 expression and induces dectin-1-dependent reactive oxygen species (ROS) generation. Immunobiology 214, 321–330, 10.1016/j.imbio.2008.09.002 (2009). 19327548

[b21] Wernersson S. & Pejler G. Mast cell secretory granules: armed for battle. Nat Rev Immunol 14, 478–494, 10.1038/nri3690 (2014). 24903914

[b22] Maurer M. *et al.* Skin mast cells control T cell-dependent host defense in Leishmania major infections. FASEB J 20, 2460–2467, 10.1096/fj.06-5860com (2006). 17142795

[b23] Echtenacher B., Mannel D. N. & Hultner L. Critical protective role of mast cells in a model of acute septic peritonitis. Nature 381, 75–77, 10.1038/381075a0 (1996). 8609992

[b24] Fukuishi N. *et al.* Does beta-Hexosaminidase Function Only as a Degranulation Indicator in Mast Cells? The Primary Role of beta-Hexosaminidase in Mast Cell Granules. J Immunol 193, 1886–1894, 10.4049/jimmunol.1302520 (2014). 25015817

[b25] Caughey G. H. Mast cell tryptases and chymases in inflammation and host defense. Immunol Rev 217, 141–154, 10.1111/j.1600-065X.2007.00509.x (2007). 17498057 PMC2275918

[b26] Oh S. W. *et al.* Tryptase inhibition blocks airway inflammation in a mouse asthma model. J Immunol 168, 1992–2000 (2002). 11823536 10.4049/jimmunol.168.4.1992

[b27] Marshall J. S. Mast-cell responses to pathogens. Nat Rev Immunol 4, 787–799, 10.1038/nri1460 (2004). 15459670

[b28] Shin K. *et al.* Mouse mast cell tryptase mMCP-6 is a critical link between adaptive and innate immunity in the chronic phase of Trichinella spiralis infection. J Immunol 180, 4885–4891 (2008). 18354212 10.4049/jimmunol.180.7.4885 PMC2969178

[b29] Thakurdas S. M. *et al.* The mast cell-restricted tryptase mMCP-6 has a critical immunoprotective role in bacterial infections. J Biol Chem 282, 20809–20815, 10.1074/jbc.M611842200 (2007). 17456473

[b30] Wang B. *et al.* Cutting edge: Deficiency of macrophage migration inhibitory factor impairs murine airway allergic responses. J Immunol 177, 5779–5784 (2006). 17056501 10.4049/jimmunol.177.9.5779

[b31] Rumsaeng V. *et al.* Human mast cells produce the CD4+ T lymphocyte chemoattractant factor, IL-16. J Immunol 159, 2904–2910 (1997). 9300714

[b32] Hosseinzadeh A., Messer P. K. & Urban C. F. Stable Redox-Cycling Nitroxide Tempol Inhibits NET Formation. Front Immunol 3, 391, 10.3389/fimmu.2012.00391 (2012). 23269921 PMC3529397

[b33] Lin A. M. *et al.* Mast cells and neutrophils release IL-17 through extracellular trap formation in psoriasis. J Immunol 187, 490–500, 10.4049/jimmunol.1100123 (2011). 21606249 PMC3119764

[b34] Scheb-Wetzel M., Rohde M., Bravo A. & Goldmann O. New insights into the antimicrobial effect of mast cells against Enterococcus faecalis. Infect Immun. 10.1128/IAI.02114-14 (2014). PMC4249337 25114115

[b35] Guery B. P. *et al.* Management of invasive candidiasis and candidemia in adult non-neutropenic intensive care unit patients: Part I. Epidemiology and diagnosis. Intensive Care Med 35, 55–62, 10.1007/s00134-008-1338-7 (2009). 18972101

[b36] Trevisan E. *et al.* Mast Cells Kill Candida albicans in the Extracellular Environment but Spare Ingested Fungi from Death. Inflammation. 10.1007/s10753-014-9951-9 (2014). 24950781

[b37] Sakurai A., Yamaguchi N. & Sonoyama K. Cell Wall Polysaccharides of Candida albicans Induce Mast Cell Degranulation in the Gut. Biosci Microbiota Food Health 31, 67–70, 10.12938/bmfh.31.67 (2012). 24936351 PMC4034280

[b38] Dongari-Bagtzoglou A. & Kashleva H. Candida albicans triggers interleukin-8 secretion by oral epithelial cells. Microb Pathog 34, 169–177 (2003). 12668140 10.1016/s0882-4010(03)00004-4

[b39] Schramm R., Schaefer T., Menger M. D. & Thorlacius H. Acute mast cell-dependent neutrophil recruitment in the skin is mediated by KC and LFA-1: inhibitory mechanisms of dexamethasone. J Leukoc Biol 72, 1122–1132 (2002). 12488493

[b40] De Filippo K. *et al.* Mast cell and macrophage chemokines CXCL1/CXCL2 control the early stage of neutrophil recruitment during tissue inflammation. Blood 121, 4930–4937, 10.1182/blood-2013-02-486217 (2013). 23645836

[b41] Pimentel T. A., Sampaio A. L., D’Acquisto F., Perretti M. & Oliani S. M. An essential role for mast cells as modulators of neutrophils influx in collagen-induced arthritis in the mouse. Lab Invest 91, 33–42, 10.1038/labinvest.2010.140 (2011). 20714326 PMC3498880

[b42] Weber F. C. *et al.* Neutrophils are required for both the sensitization and elicitation phase of contact hypersensitivity. J Exp Med 212, 15–22, 10.1084/jem.20130062 (2015). 25512469 PMC4291534

[b43] Butler J. T., Samantaray S., Beeson C. C., Ray S. K. & Banik N. L. Involvement of calpain in the process of Jurkat T cell chemotaxis. J Neurosci Res 87, 626–635, 10.1002/jnr.21882 (2009). 18831007 PMC2678561

[b44] Noverr M. C., Noggle R. M., Toews G. B. & Huffnagle G. B. Role of antibiotics and fungal microbiota in driving pulmonary allergic responses. Infect Immun 72, 4996–5003, 10.1128/IAI.72.9.4996-5003.2004 (2004). 15321991 PMC517468

[b45] Brinkmann V. *et al.* Neutrophil extracellular traps kill bacteria. Science 303, 1532–1535, 10.1126/science.1092385 (2004). 15001782

[b46] Simon D. *et al.* Eosinophil extracellular DNA traps in skin diseases. J Allergy Clin Immunol 127, 194–199, 10.1016/j.jaci.2010.11.002 (2011). 21211654

[b47] Mohanan S., Horibata S., McElwee J. L., Dannenberg A. J. & Coonrod S. A. Identification of macrophage extracellular trap-like structures in mammary gland adipose tissue: a preliminary study. Front Immunol 4, 67, 10.3389/fimmu.2013.00067 (2013). 23508122 PMC3600535

[b48] Morshed M. *et al.* NADPH Oxidase-Independent Formation of Extracellular DNA Traps by Basophils. J Immuno. 10.4049/jimmunol.1303418 (2014). 24771850

[b49] Moretti S. *et al.* The contribution of PARs to inflammation and immunity to fungi. Mucosal Immunol 1, 156–168, 10.1038/mi.2007.13 (2008). 19079173

[b50] Zhu W. & Filler S. G. Interactions of Candida albicans with epithelial cells. Cell Microbiol 12, 273–282, 10.1111/j.1462-5822.2009.01412.x (2010). 19919567 PMC3383095

[b51] O’Meara T. R. *et al.* Global analysis of fungal morphology exposes mechanisms of host cell escape. Nat Commun 6, 6741, 10.1038/ncomms7741 (2015). 25824284 PMC4382923

[b52] Uwamahoro N. *et al.* The pathogen Candida albicans hijacks pyroptosis for escape from macrophages. MBio 5, e00003–00014, 10.1128/mBio.00003-14 (2014). 24667705 PMC3977349

[b53] McAlpine S. M., Enoksson M., Lunderius-Andersson C. & Nilsson G. The effect of bacterial, viral and fungal infection on mast cell reactivity in the allergic setting. J Innate Immun 3, 120–130, 10.1159/000323350 (2011). 21242671

[b54] Gillum A. M., Tsay E. Y. & Kirsch D. R. Isolation of the Candida albicans gene for orotidine-5’-phosphate decarboxylase by complementation of S. cerevisiae ura3 and E. coli pyrF mutations. Mol Gen Genet 198, 179–182 (1984). 6394964 10.1007/BF00328721

[b55] Gerami-Nejad M., Dulmage K. & Berman J. Additional cassettes for epitope and fluorescent fusion proteins in Candida albicans. Yeast 26, 399–406, 10.1002/yea.1674 (2009). 19504625 PMC3086567

[b56] Care R. S., Trevethick J., Binley K. M. & Sudbery P. E. The MET3 promoter: a new tool for Candida albicans molecular genetics. Mol Microbiol 34, 792–798 (1999). 10564518 10.1046/j.1365-2958.1999.01641.x

[b57] Fonzi W. A. & Irwin M. Y. Isogenic strain construction and gene mapping in Candida albicans. Genetics 134, 717–728 (1993). 8349105 10.1093/genetics/134.3.717 PMC1205510

[b58] Staab J. F., Bahn Y. S. & Sundstrom P. Integrative, multifunctional plasmids for hypha-specific or constitutive expression of green fluorescent protein in Candida albicans. Microbiology 149, 2977–2986 (2003). 14523129 10.1099/mic.0.26445-0

[b59] Butterfield J. H., Weiler D., Dewald G. & Gleich G. J. Establishment of an immature mast cell line from a patient with mast cell leukemia. Leuk Res 12, 345–355 (1988). 3131594 10.1016/0145-2126(88)90050-1

[b60] Xiang Z., Moller C. & Nilsson G. IgE-receptor activation induces survival and Bfl-1 expression in human mast cells but not basophils. Allergy 61, 1040–1046, 10.1111/j.1398-9995.2006.01024.x (2006). 16918505

[b61] Ekoff M., Moller C., Xiang Z. & Nilsson G. Coaggregation of FcepsilonRI with FcgammaRIIB Inhibits Degranulation but Not Induction of Bcl-2 Family Members A1 and Bim in Mast Cells. Allergy Asthma Clin Immunol 2, 87–97, 10.1186/1710-1492-2-3-87 (2006). 20525153 PMC2876181

[b62] Stylianou M. *et al.* Antifungal application of nonantifungal drugs. Antimicrob Agents Chemother 58, 1055–1062, 10.1128/AAC.01087-13 (2014). 24277040 PMC3910836

