# Supplementary material for: Opportunistic pathogen Candida albicans elicits a temporal response in primary human mast cells
Source: Sci Rep. 2015 Jul 20;5:12287. doi: 10.1038/srep12287 (PMC4507480; doi:10.1038/srep12287)
Supplement: Supplementary Information [file srep12287-s1.pdf]

Supplementary information

Title: Opportunistic pathogen *Candida albicans* elicits a temporal response in primary human mast cells.

Running title: Human mast cells and *Candida albicans*

José Pedro Lopes[1,2,3] , Marios Stylianou[1,2,3], Gunnar Nilsson[4], Constantin F. Urban[1,2,3,#]

J.P.L and M.S. contributed equally to this work

[1]Department of Clinical Microbiology, Umeå University, Umeå, Sweden

[2] Umeå Centre for Microbial Research (UCMR)

[3]The Laboratory for Molecular Infection Medicine Sweden (MIMS)

[4]Department of Medicine, Karolinska Institutet and Karolinska University Hospital, Stockholm, Sweden

Keywords: mycoses, mast cells, *Candida albicans,* innate immunity, immune evasion, neutrophils

*** Corresponding author**

Constantin F. Urban: constantin.urban@umu.se

Supplementary Movie S1: Shown is a three-dimensional view of figure 3A, assembled by Z-stacking of micrographs from *C. albicans*-infected HMC-1 (MOI 0.1). Captured images show MCET structures in close proximity to the fungal surface trapping the microbe. Brightness and contrast were adjusted by even application to the whole field using Bitplane Imaris.

Supplementary Movie S2: *C. albicans* strain (*CAI4 pENO1-GFP-CyC1t*) constitutively expressing GFP was used to infect HMC-1 cells (MOI 1). For each experiment at least 2 microscopy fields were monitored for 16 h. *C. albicans* yeast cells were internalized into mast cells, as early as 30 min post infection. Yeast cells were able to grow intracellularly during the first hours and *C. albicans* germination resulted in collapse of the mast cell plasma membrane (arrow).

Supplementary Movie S3: *C. albicans* strain (*CAI4 pENO1-GFP-CyC1t*) was used to infected HMC-1 (MOI 1). Intracellular yeast cells were able to grow resulting in collapse of the mast cell plasma membrane comparable to Movie S2 (arrow). In a similar manner, a hypha growing in the extracellular environment and nudging a mast cell was to induce cell collapse (asterisk).

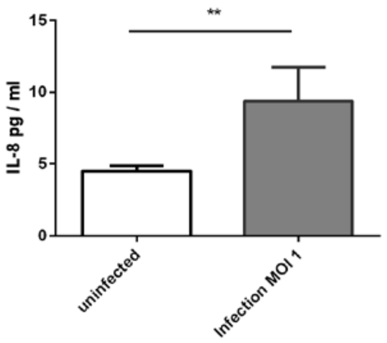

Supplementary Figure S1: Cord blood-derived mast cells released IL-8 upon *C. albicans* infection. Cord-blood derived mast cells were infected with *C. albicans* (MOI 0.1 and 1) or left uninfected. After 6 h the supernatants of infections were collected and IL-8 was measured using an ELISA. Primary mast cells release IL-8 in a similar range as HMC-1 cell line upon infection. A representative result out of two is shown as technical replicates in triplicate and analysed by t-test with Welch correction ±SD.

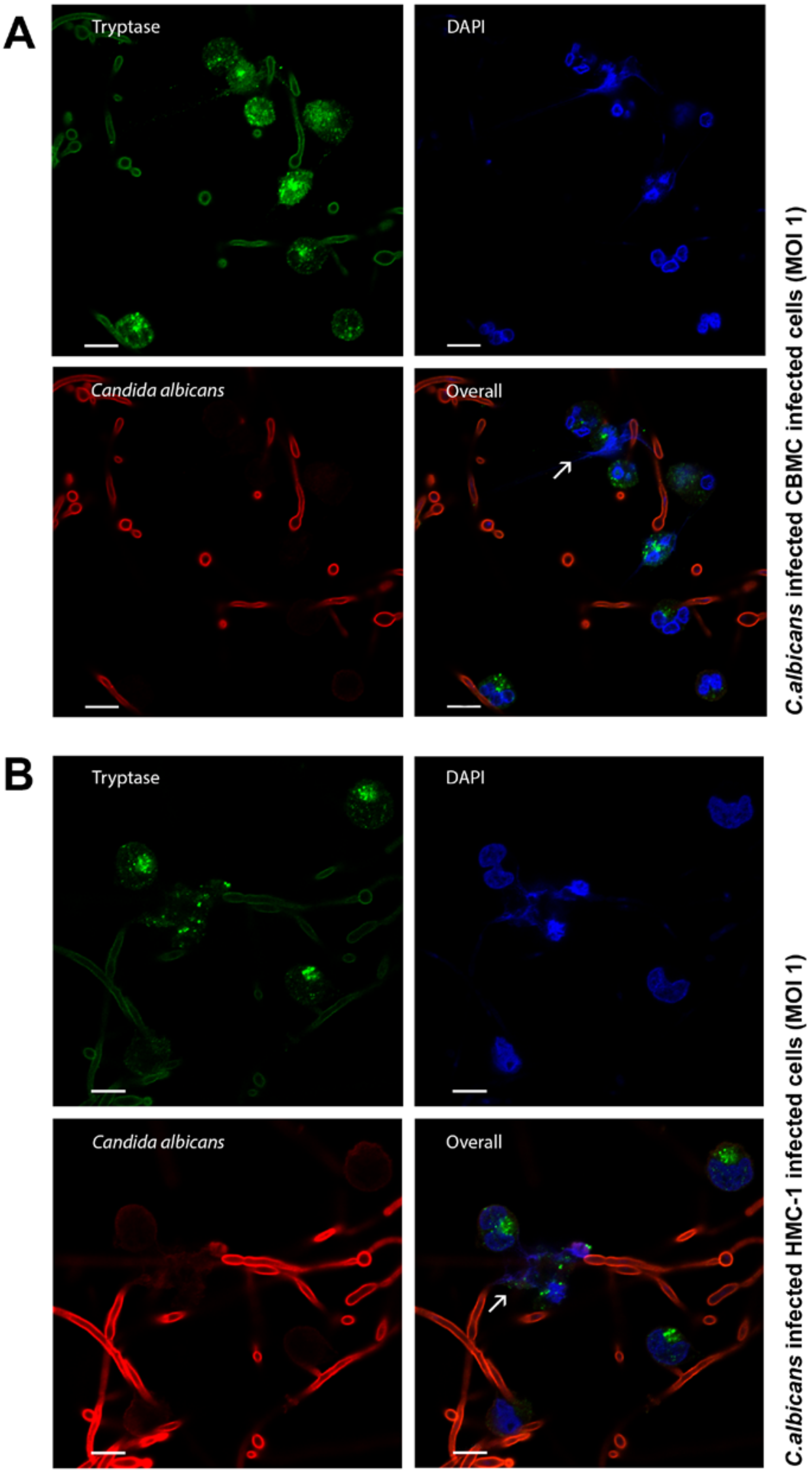

Supplementary Figure S2: Infection with higher MOIs of *C. albicans* induced MCETs in cord blood-derived mast cells and HMC-1 cells but not a substantial increase in their number. Cord-blood derived mast cells (A) and HMC-1 (B) were infected for 6 h with *C. albicans* (MOI 1). Immunostaining was performed for DNA

(DAPI, blue) and mast cell tryptase (anti-tryptase antibody followed by secondary antibody coupled to Alexa 488, green) as well as *C. albicans* (anti-*C. albicans* antibody, followed by secondary antibody coupled to Alexa 568, red). MCETs were identified by co -localization of extracellular laminar DNA and tryptase (arrows). For cord blood-derived mast cells one out of two independent experiments (from unrelated donors) is shown. Scale bars, 10 µm.

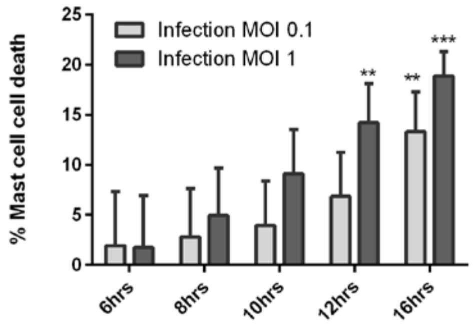

Supplementary Figure S3: *C. albicans* induced cellular death in cord blood-derived mast cells. *C. albicans* induced cell death in cord blood-derived mast cells in a time and dose-dependent manner. The Y-axis represents the relative amount of dead cells after normalization to the mast cell lysis control. Significance was analysed by Bonferroni two-way ANOVA *P≤ 0.05 comparing to the mast cell uninfected control at each time point. Cord blood-derived mast cells were differentiated from 3 independent preparations. Data is presented as n= 5 (5) ±SD.
